# Supplementary material for: Human exposure to zoonotic malaria vectors in village, farm and forest habitats in Sabah, Malaysian Borneo
Source: PLoS Negl Trop Dis. 2020 Sep 4;14(9):e0008617. doi: 10.1371/journal.pntd.0008617 (PMC7497982; doi:10.1371/journal.pntd.0008617)
Supplement: S1 Table — Characteristics of the eleven villages in Sabah State, Malaysia, where mosquito vectors were sampled. “Crops” describes the dominant types of subsistence farming occurring in the village. “Approximate area of forest patch” refers to the size of the forest patch (estimated from map) in which mosquito collections were conducted within the forest habitat type. “Population size” refers to the estimated number of residents derived from household enumeration conducted as part of the Monkeybar cross-sectional survey in September to December 2015. (DOCX) [file pntd.0008617.s001.docx]

**Table S1.**

| **District** | **Village** | **Elevation**  **(m)** | **Crops** | **Approximate area of forest patch (m^2^)** | **Population** | **Date of first sampling night** |
| --- | --- | --- | --- | --- | --- | --- |
| Kudat | Barankason (BAR) | 128 | Rubber | NA | 84 | 23/05/16 |
| Kudat | Sungai Pupu (SUN) | 57 | Rubber | 10 000 | 75 | 19/04/16 |
| Kudat | Suvil (SUV) | 9 | Rubber, palm | 500 | 77 | 25/04/16 |
| Kota Marudu | Kotud (KOT) | 543 | Rubber, palm | 75 | 245 | 30/05/16 |
| Kota Marudu | Patiu (PAT) | 260 | Rubber | 250 000 | 231 | 03/05/16 |
| Kota Marudu | Sorinsim (SOR) | 180 | Rubber | 10 000 000 | 158 | 05/04/16 |
| Pitas | Perpaduan (PER) | 14 | Palm | 1 600 | 284 | 09/05/16 |
| Pitas | Sinangip (SIN) | 218 | Rubber, palm | 500 | 375 | 11/04/16 |
| Ranau | Gondohon (GON) | 1275 | Rubber, cabbage | 500 000 | 460 | 13/06/16 |
| Ranau | Lipasu Lama (LIP) | 897 | NA | 250 000 | 311 | 16/05/16 |
| Ranau | Siba Bundu Tuhan (SIB) | 1084 | Cabbage, lettuce | 10 000 000 | 737 | 21/03/16 |
