# Supplementary material for: Human exposure to zoonotic malaria vectors in village, farm and forest habitats in Sabah, Malaysian Borneo
Source: PLoS Negl Trop Dis. 2020 Sep 4;14(9):e0008617. doi: 10.1371/journal.pntd.0008617 (PMC7497982; doi:10.1371/journal.pntd.0008617)
Supplement: S2 Table — (DOCX) [file pntd.0008617.s002.docx]

| **Target** | **PCR** | **Primer** | **Sequence (5’ - 3’)** | **Annealing temp.** | **Product size** |
| --- | --- | --- | --- | --- | --- |
| *Plasmodium* genus | 1/ 2 | rPLU1 | TCAAAGATTAAGCCATGCAAGTGA | 55^o^C | 1.6-1.7kb |
|  |  | rPLU5 | CCTGTTGTTGCCTTAAACTCC |  |  |
| *Plasmodium* genus | 1 | rPLU3 | TTTTTATAAGGATAACTACGGAAAAGCTGT | 62^o^C | 235bp |
|  |  | rPLU4 | TACCCGTCATAGCCATGTTAGGCCAATACC |  |  |
| *P. coatneyi* | 2 | PctF1 | CGCTTTTAGCTTAAATCCACATAACAGAC | 62^o^C | 504bp |
|  |  | PctR1 | GAGTCCTAACCCCGAAGGGAAAGG |  |  |
| *P. inui* | 2 | PinF2 | CGTATCGACTTTGTGGCATTTTTCTAC | 60^o^C | 479bp |
|  |  | INAR3 | GCAATCTAAGAGTTTTAACTCCTC |  |  |
| *P. fieldi* | 2 | PfldF1 | GGTCTTTTTTTTGCTTCGGTAATTA | 66^o^C | 421bp |
|  |  | PfldR2 | AGGCACTGAAGGAAGCAATCTAAGAGTTTC |  |  |
| *P. cynomolgi* | 2 | CY2F | GATTTGCTAAATTGCGGTCG | 60^o^C | 137bp |
|  |  | CY4R | CGGTATGATAAGCCAGGGAAGT |  |  |
| *P. knowlesi* | 2 | PkF1140 | GATTCATCTATTAAAAATTTGCTTC | 50^o^C | 424bp |
|  |  | PkR1550 | GAGTTCTAATCTCCGGAGAGAAAAGA |  |  |
| *P. falciparum* | 2 | NewPLFshort | CTATCAGCTTTTGATGTTAG | 53^o^C | 370bp |
|  |  | FARshort | GTTCCCCTAGAATAGTTACA |  |  |

**Table S2.**

Table S2. continued on next page

| **Target** | **PCR** | **Primer** | **Sequence (5’ - 3’)** | **Annealing Temp.** | **Product size** |
| --- | --- | --- | --- | --- | --- |
| *P. vivax* | 2 | NewPLFshort | CTATCAGCTTTTGATGTTAG | 53^o^C | 476bp |
|  |  | VIRshort | AAGGACTTCCAAGCC |  |  |
| *P. malariae* | 2 | NewPLFshort | CTATCAGCTTTTGATGTTAG | 53^o^C | 241bp |
|  |  | MARshort | TCCAATTGCCTTCTG |  |  |
| *P. ovale* | 2 | NewPLFshort | CTATCAGCTTTTGATGTTAG | 53^o^C | 407bp |
|  |  | OVRshort | AGGAATGCAAAGARCAG |  |  |

**Table S2 continued.**
