# Supplementary material for: Human exposure to zoonotic malaria vectors in village, farm and forest habitats in Sabah, Malaysian Borneo
Source: PLoS Negl Trop Dis. 2020 Sep 4;14(9):e0008617. doi: 10.1371/journal.pntd.0008617 (PMC7497982; doi:10.1371/journal.pntd.0008617)
Supplement: S3 Table — (DOCX) [file pntd.0008617.s003.docx]

**Table S3.**

|  | **District of sampling** | | | | | | | | | | |  |
| --- | --- | --- | --- | --- | --- | --- | --- | --- | --- | --- | --- | --- |
|  | **Kudat (villages)** | | | **Kota Marudu (villages)** | | | **Pitas (villages)** | | **Ranau (villages)** | | |  |
| **Mosquito genera** | **SUV** | **SUN** | **BAR** | **SOR** | **PAT** | **KOT** | **PER** | **SIN** | **LIP** | **SIB** | **GON** | **Total (%)** |
| *Aedes* sp. | 6 | 17 | 14 | 20 | 46 | 72 | 86 | 56 | 19 | 34 | 54 | 424 (7.6) |
| *Anopheles* sp. | 3 | 34 | 1 | 34 | 8 | 33 | 2 | 43 | 19 | 31 | 4 | 212 (3.8) |
| *Armigeres* sp. | 162 | 64 | 313 | 124 | 19 | 97 | 581 | 612 | 34 | 14 | 1 | 2021 (36.2) |
| *Culex* sp. | 115 | 78 | 1663 | 55 | 118 | 354 | 172 | 16 | 54 | 142 | 14 | 2781 (49.8) |
| *Mansonia* sp. | 46 | 0 | 0 | 0 | 2 | 0 | 31 | 0 | 3 | 0 | 0 | 82 (1.5) |
| *Orthopodomyia* sp. | 0 | 0 | 0 | 0 | 0 | 0 | 3 | 0 | 0 | 0 | 0 | 3 (0.1) |
| *Uranotaenia* sp. | 0 | 0 | 0 | 1 | 0 | 0 | 0 | 9 | 0 | 0 | 0 | 10 (0.2) |
| *Verrallina* sp. | 0 | 0 | 2 | 0 | 0 | 0 | 41 | 0 | 0 | 0 | 0 | 43 (0.8) |
| Unknown | 2 | 1 | 2 | 0 | 1 | 1 | 2 | 1 | 0 | 2 | 0 | 12 (0.2) |
| Total | 334 | 194 | 1995 | 234 | 194 | 557 | 918 | 737 | 129 | 223 | 73 | 5588 |
