# Supplementary material for: Human exposure to zoonotic malaria vectors in village, farm and forest habitats in Sabah, Malaysian Borneo
Source: PLoS Negl Trop Dis. 2020 Sep 4;14(9):e0008617. doi: 10.1371/journal.pntd.0008617 (PMC7497982; doi:10.1371/journal.pntd.0008617)
Supplement: S4 Table — (DOCX) [file pntd.0008617.s004.docx]

**Table S4.**

| **Habitat** | **Mean elevation (m) (range)** | **Predicted mean elevation (m) (range)** | **Tukey’s test between means** | **Mean percentage forest cover (%) (range)** | **Predicted mean percentage forest cover (range)** | **Tukey’s test between means** |
| --- | --- | --- | --- | --- | --- | --- |
| Peri-domestic (PD) | 427.1 (14 – 1109) | 196.5 (128.4 – 300.7) | PD – FA, *P* = 0.34 | 10.2 (0 – 70.5) | 3.8 (0.8 – 15.3) | PD – FA, *P* = 0.58 |
| Farm (FA) | 358.5 (13 – 1107) | 193.3 (126.4 – 295.8) | FA – FO, *P* = 0.71 | 17.3 (0 – 62.5) | 14.3 (6.1 – 30.0) | FA – FO, *P* = 0.74 |
| Forest (FO) | 478.1 (15 – 1125) | 195.1 (127.5 – 298.7) | FO – PD, *P* = 0.79 | 24.2 (0 – 61.4) | 8.8 (2.8 – 24.2) | FO – PD, *P* = 0.23 |
