# Supplementary material for: Human exposure to zoonotic malaria vectors in village, farm and forest habitats in Sabah, Malaysian Borneo
Source: PLoS Negl Trop Dis. 2020 Sep 4;14(9):e0008617. doi: 10.1371/journal.pntd.0008617 (PMC7497982; doi:10.1371/journal.pntd.0008617)
Supplement: S5 Table — GLMs were used to compute (R2 and effect sizes) for subsequent power analyses to determine sample sizes required to generate the same associations (P = 0.05) with 80% power. (DOCX) [file pntd.0008617.s005.docx]

**Table S5.**

|  | **Dependent variable (coefficient)** | **Estimate from GLM** | **AIC** | **R^2^** | **Effect size (R^2^ – (1-R^2^))** | **Sample size for *P* = 0.05, power = 80%** |
| --- | --- | --- | --- | --- | --- | --- |
| Model 1 | Probability of trapping *An. balabacensis* per night | 1.156 (*P* = 0.8850) | 5.3754 | 0.0530 | 0.0560 | 142 villages |
| Model 2 | Probability of trapping *An. Leucosphyrus gp.* per night | 1.122 (*P* = 8710) | 5.3758 | 0.0662 | 0.0710 | 390 villages |
| Model 3 | Density of *An. balabacensis* trapped per night | 0.7134 (*P* = 0.8894) | 5.3753 | 0.0475 | 0.0499 | 159 villages |
| Model 4 | Density of *An. Leucosphyrus gp.* trapped per night | 0.3938 (*P* = 0.9287) | 5.3746 | 0.0197 | 0.0201 | 113 villages |
