# Supplementary figures and images for: Human exposure to zoonotic malaria vectors in village, farm and forest habitats in Sabah, Malaysian Borneo
Source: PLoS Negl Trop Dis. 2020 Sep 4;14(9):e0008617. doi: 10.1371/journal.pntd.0008617 (PMC7497982; doi:10.1371/journal.pntd.0008617)

**Figure S1.**


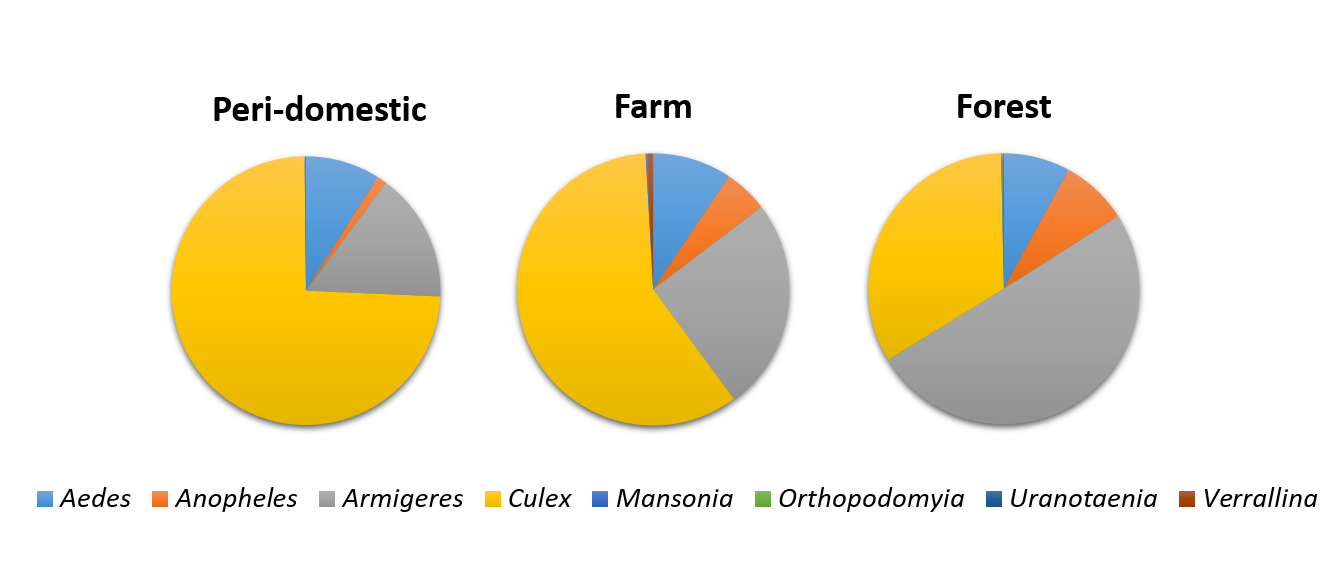

Supplement: S1 Fig — (DOCX) [file pntd.0008617.s006.docx]

**Figure S2.**


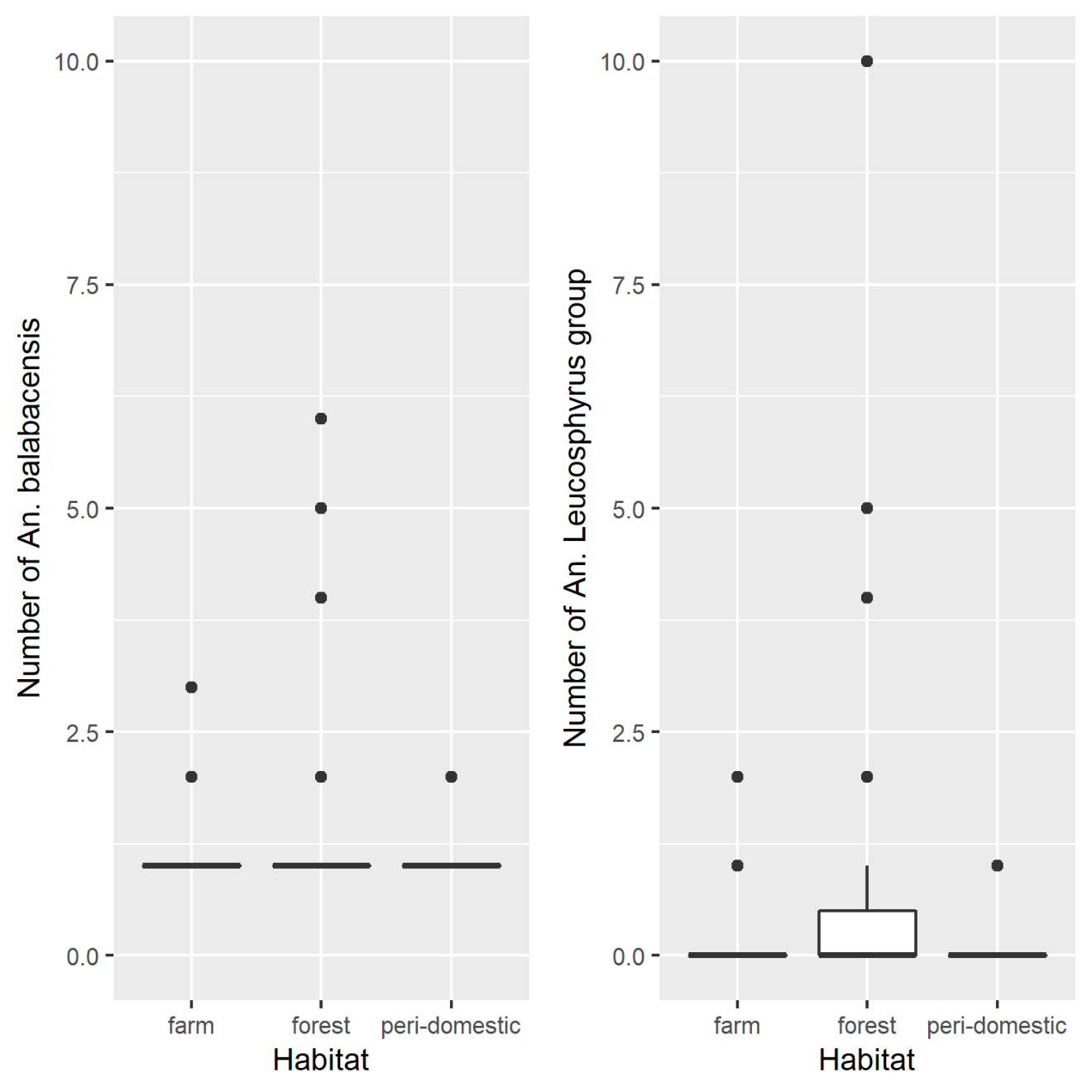

Supplement: S2 Fig — (DOCX) [file pntd.0008617.s007.docx]

**Figure S3.**


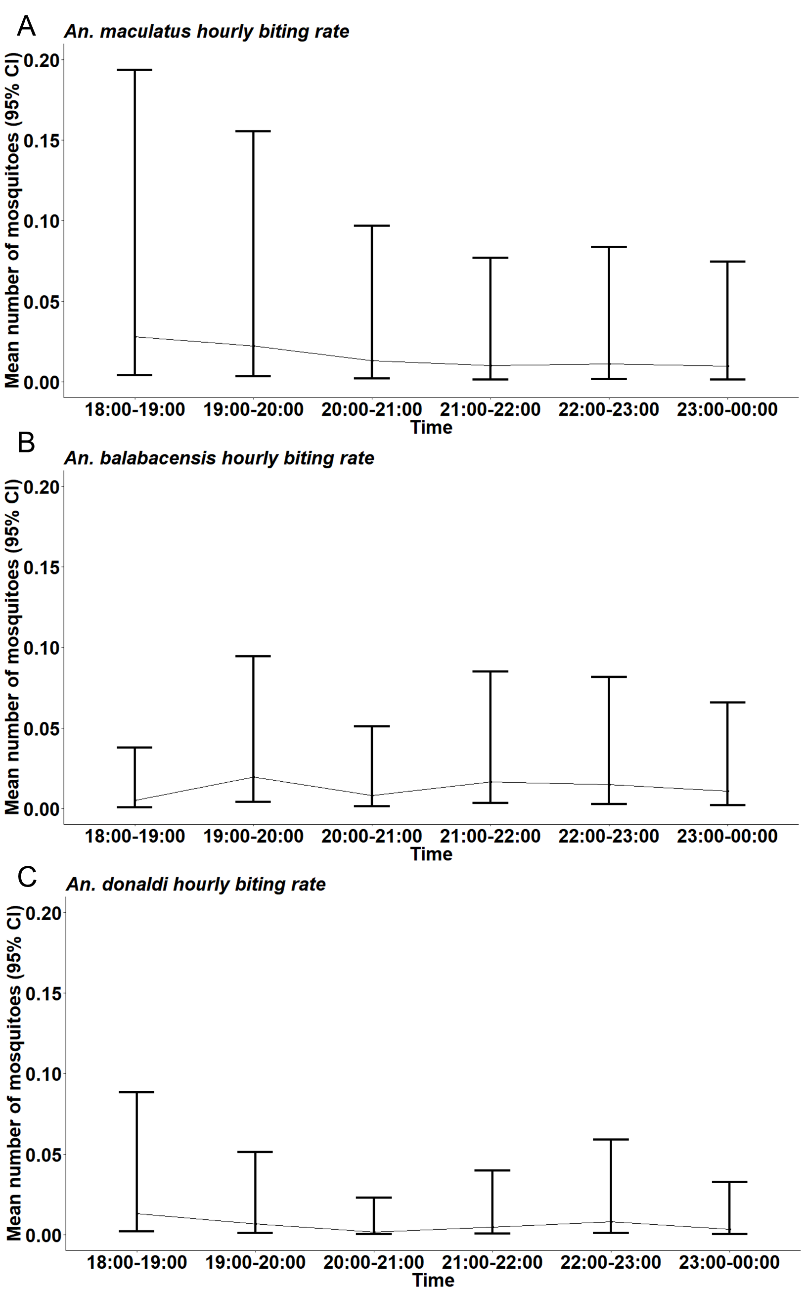

Supplement: S3 Fig — Predicted mean number of A) An. balabacensis, B) An. donaldi and C) An. maculatus biting per hour between 18:00–24:00 hrs, pooled across all sites and habitat types. Error bars are 95% confidence intervals. (DOCX) [file pntd.0008617.s008.docx]
