# Supplementary material for: Human exposure to zoonotic malaria vectors in village, farm and forest habitats in Sabah, Malaysian Borneo
Source: PLoS Negl Trop Dis. 2020 Sep 4;14(9):e0008617. doi: 10.1371/journal.pntd.0008617 (PMC7497982; doi:10.1371/journal.pntd.0008617)
Supplement: Attachment — Submitted filename: PNTD-D-19-02061.pdf [file pntd.0008617.s009.pdf]

Please find in the following my comments on the manuscript titled, "Human exposure to zoonotic malaria vectors in village, farm and forest habitats in Sabah, Malaysian Borneo" by Brown et al. This manuscript is trying to find out whether data obtained on vector densities and habitats from small areas can be generalized for larger areas.

General comments

The message of this manuscript is not clear because the introduction and discussion sections are long, and results are contradicting the group's previous findings and arguing something which is not very convincing. The main findings in this study are that the density of *An. balabacensis* was much lower and vector densities were higher in farm and forest habitats compared with previous studies. Previous studies found vector abundance in the peridomestic settings. My concern is what is the new finding in this manuscript? It is well known that the data obtained from small areas cannot be extrapolated in large area due to the presence of several variables. There is a time difference between this study and the previous studies, which might have a considerable effect on the results. Certainly, the authors expressed this concern in the manuscript, but it cannot prevent readers from wondering which results are correct, the previous or the present findings.

Day by day the forest areas are shrinking in Sabah, monkeys are losing their habitats and insecticides are used abundantly to control mosquitoes. Therefore, time is a major factor in this type of results from Sabah.

Specific comments

1. Author summary; line 57: *P. knowlesi* is not recently spilled over into human rather it is now an established cause of malaria in this part of the world.
2. The introduction and discussion sections should be shortened, clear and to the point. I think then it will be easy for international readers.
3. There were no comments on *An. donaldi* and *An. lateus* which are vectors of *P. knowlesi* (Hanks et al. 2019; Chua et al. 2019).
4. A yearlong study found that *An. balabacensis* is equally abundant in different habitats (Chua et al 2019). I think a comparison with this study will be helpful to understand.
5. Lines 131–138: There are several factors, such as types of agriculture, availability of mosquito breeding sites, land cover might affect the mosquito density and types. However, presence of macaques in the study area might affect the vectors specifically vectors containing *P. knowlesi.* I think data on the presence of macaques in the study areas should be added.
6. The results presented here is the cumulative data obtained from 4 districts, what about the data from Kudat only. Are the results only from Kudat similar as obtained in previous studies?
7. Line 267: How Plasmodium infection was determined in mosquito by microscopy?
8. Table 1: Indicate the full name of each village.
9. Were there any macaque habitats near the study sites?
10. Lines 446-447: What do you mean by this sentence?
11. Line 463: Why putative?
12. The method used to determine the serology is not clear. Which antigens were used in the present study? One of my main concern in this manuscript is whether this method was validated

to determine the specificity of infection at population level? Proper references are needed in this regard.
