# Supplementary material for: Human exposure to zoonotic malaria vectors in village, farm and forest habitats in Sabah, Malaysian Borneo
Source: PLoS Negl Trop Dis. 2020 Sep 4;14(9):e0008617. doi: 10.1371/journal.pntd.0008617 (PMC7497982; doi:10.1371/journal.pntd.0008617)
Supplement: Attachment — Submitted filename: PLOSntds reviewers comments addressed.pdf [file pntd.0008617.s010.pdf]

Thank you for your comments on our submission **'Human exposure to zoonotic malaria vectors in village, farm and forest habitats in Sabah, Malaysian Borneo'** to PLOSntds. Please find below a summary (duplication removed) of all reviewer's comments addressed by the authors. All references to line numbers relate to the tracked changes version of the manuscript.

#Reviewer 1

Reviewer 1: All sites were studied in three months (March to June 2016) the single time frame did not seem to particularly coincide with annual zoonotic malaria prevalence in Sabah. Even-so to attempt sampling 3 habitats, with teams of two per habitat, using only human landing catches (HLC) in 11 villages over a 3-month period would be expected to be limiting.

Author: In contrast to seasonal malaria transmission patterns observed in other parts of the world, there is no peak season for *P. knowlesi* transmission in Malaysian Borneo, with both rainfall and reported malaria cases varying monthly and yearly. Longitudinal surveys have shown that vector densities remain relatively constant throughout the year, irrespective of sampling month thus no strong seasonal pattern of vector abundance has been detected in Sabah (1,2).

However, the reviewer raises an important point that these results are limited temporally with sampling restricted to a 3 month period. This was based on a strategic decision to reduce the temporal coverage of sampling so as to maximize spatial coverage. Ideally it would be possible to measure human exposure to mosquitoes both for a long period (e.g a year) and a large number of sites. However, the logistics and high expense meant it was not possible to achieve such high resolution spatial and temporal sampling. Previous studies have already investigated temporal variability in *P. knowlesi* exposure (and shown little seasonality (1)), but only at a small number of sites in a limited geographic area. Thus our primary focus was on spatial heterogeneity. This study measured mosquito biting rates over a much wider spatial area and range of habitat types than previous work, providing valuable information about mosquito ecology within this larger region. Thus while we acknowledge a longer period of sampling would have been desirable, we believe the insights gained over this 3 month period are appropriate to give a snap shot of spatial heterogeneity in vector density and diversity.

Reviewer 1: Sampling was conducted between 6pm and midnight when 6pm to 6am would have been more comprehensive.

Author: This sampling design was based on previous studies within this region where the primary vector *An. balabacensis* was sampled using human landing catches between 6pm and 6am. Here the majority of biting took place before midnight (2,3). Additionally, the number of *An. balabacensis* caught during all night catches is highly correlated with catches from 6pm- midnight (see Fornace *et. al*, *ELife* (2), comparison of these numbers from this reference included below). Given the relatively minor degree of biting that takes place outside of 6pm-12am, and the fact that few people are outside of their homes during such late-night hours, we believe sampling between 6pm-12am was appropriate. For clarity, we have included an explanation of why this was done alongside the references in the manuscript on **line 249**.

Total *An. balabacensis* caught during 6 hour catches vs. 12 hour catches and fitted linear model (R^2= 0.85)

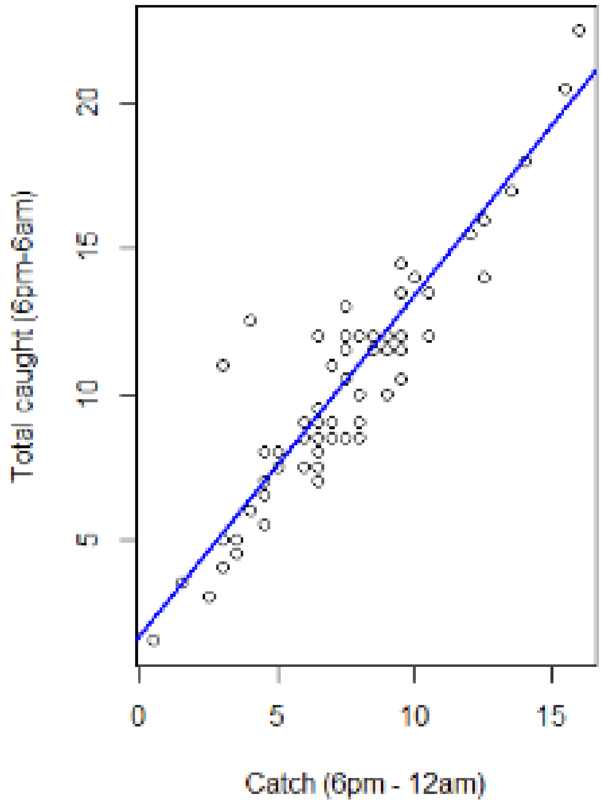

Reviewer 1: The study design would not be expected to produce the information required to address the main research questions. At best all that can be concluded is that the sampling methods used over short time intervals may not have been sufficient to properly capture the information required to calculate the environmental determinants of vector density and infection rate at the study sites during a single short time interval.

Author: As detailed above, while we agree a longer period of sampling would have been desirable, we believe the mosquito sampling design employed here is appropriate to give a general estimate of how mosquito vector densities vary spatially and between different habitats because of the limited evidence of seasonality in this area. Characterizing wider spatial heterogeneity in vector density and its relationship with habitat type was our primary aim; rather than a detailed investigation of (other) potential environmental determinants.

Additonally, the mosquito sampling method used is known to be most effective for measuring *P. knowlesi* vectors in Malaysia. Previous work by our team and others has evaluated a range of sampling methods for *P. knowlesi* vectors including light traps, resting traps and e-nets (4–6), and none have been nearly as effective as the Human Landing Catch approach used here. We agree with the reviewer that the sample size of *An. balabacensis* was too small for estimation of how vector infection rate differs with environmental variability. Given the generally low abundance of vectors through the wider region – an important new finding in itself – acquiring adequate sample sizes to test for variation infection rates could take years and is beyond the feasibility of this project. The

sample sizes obtained were sufficient to detect habitat-dependent variation in human exposure to the primary vector *An. balabacensis,* thereby fulfilling a key aim. Additionally, we highlighted wider spatial variation in vector density, encompassing districts that differed substantially ie. in elevation, tree species and climatic factors. We believe this provides novel insights into the ecology and associated human exposure of *P. knowlesi* vectors that have not been addressed in previous work. Finally, this study provides insight into trade-offs between 'depth' (sampling a couple of sites intensively) and 'breadth' (sampling more sites over a shorter period) which is a common consideration in designing vector surveillance. We have now commented on this from **line 536**.

Reviewer 1: The authors do acknowledge this but yet make statements such as An. balabacensis occurred at the study sites at lower density when compared to a much more intense and prolonged study in the Kudat District of Sabah. Had the two studies been similarly rigorous then indeed a comparison could have been made.

Author: We agree with the reviewer here that we cannot make a direct comparison between density of vectors in our study and the densities obtained from a more intensive and longitudinal sampling strategy. We have changed the manuscript to soften these claims by describing the differences in the sampling strategies (eg. **line 509**) and now focus more on the novelty of the study being the first to perform wide spatial sampling of *P. knowlesi* vectors in Malaysia. To date most studies investigating *P. knowlesi* vector bionomics have focused sampling on small spatial scales and whilst this might be good for defining local patterns in vector abundance and behaviour, they may not reflect larger scales. Data from wider scale surveillance, even if temporally limited, is important particularly for guiding vector control strategies which are often implemented at regional levels.

Reviewer 1: In the absence of rigorous longitudinal sampling the objectives of this study on the environmental determinants of malaria vector density, particularly vectors of zoonotic malaria, would be difficult to test.

Author: The reviewer raises valid points about the limitations arising from the relatively short-term sampling period of this study. However as detailed more extensively above, we believe these limitations are not as severe as the reviewer anticipates (e.g due to lack of seasonality in study area), and that the wider spatial sampling we adopt still yields several novel and important findings of use to the zoonotic malaria community. We acknowledge that the limited temporal sampling restricts the ability to identify additional non-habitat environmental determinants of malaria vector density, and have modified the text to reflect that. This study provides a first snapshot of the distribution of vectors across this region representing a much wider ecological gradient compared to previous studies and highlights the heterogeneities in distribution of *P. knowlesi* vectors within this area. In the discussion (**line 536**) we comment on the differences in sampling design between this and previous studies and explain that similar longitudinal sampling is required to assess differences with previous studies conducted in the Kudat district.

Methods:

Reviewer 1: It is not possible to comment on the integrity of DNA in the samples following ethanol preservation – however given the small number of Plasmodium vectors collected during the entire study this is perhaps a moot point. All sites were studied in three months (March to June 2016) the single time frame did not seem to particularly coincide with annual zoonotic malaria prevalence in

Sabah. Even-so to attempt sampling 3 habitats, a team of two per habitat, using only HLC in 11 villages over a 3-month period is a long shot.

Author: Previous studies (1,7–10) have indicated that the storing of mosquito/plasmodium DNA in ethanol is common practice for later PCR analysis. We acknowledge that a longer sampling period would have strengthened our ability to estimate mosquito infection rates as acknowledged above, however this was not feasible within the context of this project. However, as mentioned above, in Sabah there is no seasonal variation in zoonotic malaria transmission, so our sampling did not miss a "peak" transmission season, and vector densities during the three-month period of sampling are likely representative of those at other times of year (1). Testing for associations with human prevalence was one of three objectives; the other information gained on abundance, diversity and habitat associations of vectors would make a very useful contribution to our understanding of this hard to study vector/malaria system. Although vector sampling is more limited than would have been desirable, it is a significant advance on previous work that has largely focussed on just a small number of sites in one district. Thus, this work really complements previous work and helps fit a gap about ecology of vectors outside of Kudat.

Reviewer 1: While on the face of it this may appear to be an 'intensive' study the protocol and team size assigned for sampling at each habitat per site is, to say the least minimal, and lacks any attempt to include diverse sampling methods with no opportunity to identify differences in vector density over time. Furthermore, the study is unlikely to capture enough data to calculate vector association with seroprevalence data collected six months earlier.

Author: We can appreciate the reviewers concerns however, serology has widely been applied to characterise differences in malaria transmission and has been associated with EIR and other metrics (11). We would hypothesize that high transmission areas identified by serology may have correspondingly high vector densities; and wished to investigate this here. Our team have conducted extensive investigation of alternative vector sampling methods in this study area; including resting traps, monkey-baited traps, CDC-light traps and e-nets baited with monkeys or people (5,6). From these studies, we demonstrate that the HLC method used here is by far the most effective, thus we are confident in our choice of sampling method. The reviewer highlights an important issue that these estimates of vector density may not generalise vector densities over longer time periods however it is likely that they are suitable given the little evidence of seasonal patterns of vector abundance in this context (2).

Conclusions:

Reviewer 1: Malaria transmission in Southeast Asia, particularly Malaysian Borneo, is under intense control pressure and consequently is relatively low and temporal. The authors attempt to generate information on zoonotic malaria vector density, at best difficult, but they adopt scant, short and single capture methodologies that, in the best of circumstances, would be unlikely to generate the depth, quality or quantity of data required to identify the environmental determinants of Plasmodium knowlesi vector density and infections rates across a wider spatial scale in Sabah.

Author: In Malaysian Borneo, the two common methods of vector control are insecticide-treated nets and indoor residual spraying (12,13) however these only provide protection from mosquitoes

attempting to feed on people inside houses. The primary vector of *P. knowlesi* in Sabah is *Anopheles balabacensis* which has exophilic biting behaviour (3) making these methods of control not very effective, thus it is unlikely that these control methods are responsible for the low densities of vectors seen in this area.

As stated above, there is no seasonality in vector abundance in Sabah (1,2) thus the data presented in this manuscript may be sufficient for detailing vector density and distribution across a wide region of Sabah. The study described here is of greater breadth than previous studies have attempted and contributes to the understanding of *P. knowlesi* vector ecology outside a few focal sites in Kudat which is certainly warranted given the scale of the problem. When dealing with a Neglected Tropical Disease of complex ecology, it isn't always possible to perform vector studies incorporating depth and breadth due to limited funding however it is important that the best available data is made public. We acknowledge the limitations of the study: 1) small sample size to detect infected mosquitoes; 2) small sample size to pick up associations with human serology data; 3) lack of longitudinal sampling to robustly compare habitat associations from previous studies, but we have been transparent about these and have added further detail as described in the points above.

#Reviewer 2

Reviewer 2: If possible, some kind of power analysis based on detection rates here to determine the length and coverage of sampling that might be required to conclusively evaluate if vector density can be used as a proxy for human infection risk would be useful particularly for the intended audience of PLoS NTDs.

Author: The authors thank the reviewer for this suggestion and agree that this would be useful to guide future studies. We have added a power analysis which is described on **line 363** and discussed on **line 458 and 609**.

Reviewer 2: Throughout make sure that appropriate statistical test information is reported. For example, on line 369-398. What was the outcome of the GLMM that allows you to state that there is no systemic variation in altitude across site types. On line 433-434, what are the stats that show no significant effect of time on mean predicted number of bites.

Author: Thank you for highlighting this. We have added the results of the Tukey's post hoc test to table S4 (in the supplementary information) which indicates no significant difference in altitude or forest cover between site types and referred to this in the text on **line 403 and 406**.

For all three vector species, there was no significant effect of time on the mean predicted number of bites shown by Tukey's post hoc tests. We have now added this on **line 435**.

Reviewer 2: It was not clear to me why the outcome variable for vector density was the mean number caught per site per night. If date and site were both included as explanatory variables wouldn't it allow you to directly model number of mosquitoes caught? If date and site were both included as explanatory variables wouldn't it allow you to directly model number of mosquitoes caught?

Author: Its true that if date and site were both included as explanatory variables, then it would allow you to directly model the number of mosquitoes caught, however we were interested in examining the effect of habitat to relate it to human exposure risk, not just to get a mean number of mosquitoes per day. In the GLMM we included 'date' as a random effect because we wanted to control for random variation due to sampling on different days but were not interested in finding out whether one day had significantly higher numbers than another day.

Reviewer 2: Figure 1: I would remove a and include a figure that illustrates the "substantial variation in elevation, the size, and distribution of forest areas, and local agricultural activities". Could you use the Hansen global forest cover 2014 map for this?

Author: Thank you for this suggestion however using the Hansen data set may not be the most appropriate to show variation in land use because all grids with over 50% canopy cover are classified as forest and the dataset does not differentiate between rubber and palm. For that reason we have chosen to retain the original Figure 1 and summarise village level environmental variation in SI1 as in the original submission.

Reviewer 2: Fig 3: I would recommend a box plot for this.

Author: Thank you for the suggestion to include this data in a boxplot. We decided to plot the data in a bar plot because this can show the predicted mean as obtained from the statistical model but a boxplot generally does not. However we agree there is value in showing the underlying data in a boxplot so this has now been added to the supplementary information (Figure S2).

Reviewer 2: Fig 4: I don't think this figure is needed as long as the lack of statistical significance is fully reported in the text.

Author: We have now removed figure 4 and put it in the supplementary information (Figure S3).

Reviewer 2: The authors identify the two key limitations of the study: the low numbers of malaria vectors sampled and the temporal mismatch between the human prevalence data and the vector density data. I thought they did a respectable job of pointing out these issues, but there are a few key points to be considered and pieces of information that I think the reader needs to assess the validity of the conclusions.

Author: Thank you for the observation that we did a reasonable job of discussing limitations of the study, and appreciate the suggestions for how to further clarify the limitations of our conclusions. We aimed to incorporate these in the resubmitted version as detailed in response to points below.

Reviewer 2: 1. Are there alternative explanations for the numbers of Anopheles in HLCs? Certainly, one explanation is that there are not many Anopheles. However, only one sampling technique was reported and it maybe that this technique was not appropriate for this particular setting. For example barrier screens have been used in other settings (Pollard et al. 2019, Parasites and Vectors) to collect blood fed Anopheles. Can you offer some kind of assessment of the probability that HLCs were not effective for Anopheles in this environment. Are there any larval surveys or alternative sampling that can support the HLC result? If HLCs are a valid technique (which in many instances they are) then what kind of power would you require to detect an association with serology data. I

think this is important for recommendations moving forward. Can you use this data to determine the temporal and geographic spread you might need?

Author: We agree that it would be useful to further comment on the appropriateness of our mosquito sampling methods. The HLC approach was selected based on our previous work on trap evaluation in this area which has shown HLC to be the most effective (see **line 240**). Pilot work was done to test the effectiveness of different trapping methods for host seeking (6) and resting mosquitoes (5) in this study area. Human landing catch was selected because it was by far the most the effective of those trialled. It would be interesting to try barrier screens in this setting as this has never been done before. We do not have complementary larval survey data for the areas we sampled by HLC however we can confirm that HLC is an effective method of *An. balabacensis* capture based on extensive preliminary studies including a MSc study (Hanns Ng) performed from March 2015 to January 2016. The reviewer makes a useful suggestion about the value of conducting a power analysis to assess what power we had to detect an association between vector densities and serology data, which would be useful for informing future studies. To address this we have included a power analysis (**line 363**, **458 and 609**) to identify the sample size required to find a positive association between human *P. knowlesi* seroprevalence and vector presence/density.

Reviewer 2: 2. What was the impact of El Nino during this period. I can see from the attached Fornace publication that there was drought during the serology study which may have impacted vector densities. Was this also having an effect in 2016? Might this explain the discrepancy between site types in the two studies? During drought the oviposition may have been more likely to occur in peri-domestic habitats where people were storing water?

There were widespread droughts across Sabah during the El Nino but it is difficult to determine the effects on vectors from the data which we have because there were no sites sampled consistently before, during and after the El Nino. All HLC data available is reported in (2). We have commented on this in the discussion on **line 504**.

Reviewer 2: 3. It was not clear to me exactly what the serology data indicates and this seems imperative for helping the reader assess the association between the two data sets. From what I understood serology can be used to detect current and historical infections. On line 554 it is stated that the antigens for P. knowlesi are relatively short lived, but when I looked into the citations provided, I still could not determine how short lived. The vector data is collected in 2016 and the serology in 2015. Over what period is prevalence being captured by these data? Is it anyone exposed within 6 months, a year? We expect vector abundance to fluctuate over time at relatively short time scales compared to host infection rates and it is not clear to me why would expect human prevalence rate from a previous year (potentially a previous year with drastically different environmental factors due to el nino and calculated over a long period of time) to correlate with a snapshot of vector density taken much later and in a different season. I think the authors hit on these issues in the discussion, but they need to be much stronger in their defence of this methodology.

Author: In this analysis, the serological data is being used as proxy for transmission. Active *P. knowlesi* infections exist at such low prevalence in the human population, and because there are precedents for comparing entomological estimates of risk with serological transmission (as in (11)),

serology was used in its place. The analysis of this serological data was designed to represent relatively short-term exposure (e.g. within one year) as described by Fornace et. al, 2019 (15) and this should be a broad indication of transmission within the proceeding year. However, the rapidly changing environment and climatic factors as well as the short sampling periods for this study are all plausible reasons for the lack of association. Increases in mosquito densities have not always correlated with increases in malaria risk and this could particularly be the case with knowlesi as macaque densities and disease dynamics remain relatively unknown (we have added this as a comment on **line 609**. The difference in collection time may not be as much of an issue as the reviewer thinks due to a lack of seasonality seen in vector densities.

Reviewer 2: This is a useful data set and data on vector species in this increasingly epidemiologically important region is scarce and notoriously difficult to collect. This makes the data presented here novel and significant to those currently attempting to assess a rapidly evolving transmission landscape. Overall, the vector sampling study is well designed and executed. The data are explained clearly and rigorously assessed. There are a few weaknesses in the study related to the use of serology data, but this weakness is clearly identified. With some additional evaluation of these weakness and discussion of alternative explanations for the lack of correlation observed here I feel the study will be ready for publication.

Author: We thank the reviewer for their positive remarks on the study. In the resubmitted manuscript, we aimed to address the changes proposed by including the following:

-confirmation of HLC as suitable collection technique **(line 240)**

-acknowledging that the El Nino may have contributed to low vector densities **(line 504)**

-inclusion of power analysis to demonstrate sample size required to detect associations between vector densities and human serology measures **(line 363**, **458 and 609)**

-statement on the premise for using serology data and the limitations surrounding this **(line 173 and 281)**

We hope that this will satisfy the reviewer's criteria for publication.

#Reviewer 3

Reviewer 3: General comments: The message of this manuscript is not clear because the introduction and discussion sections are long, and results are contradicting the group's previous findings and arguing something which is not very convincing. The main findings in this study are that the density of *An. balabacensis* was much lower and vector densities were higher in farm and forest habitats compared with previous studies. Previous studies found vector abundance in the peridomestic settings. My concern is what is the new finding in this manuscript? It is well known that the data obtained from small areas cannot be extrapolated in large area due to the presence of several variables. There is a time difference between this study and the previous studies, which might have a considerable effect on the results. Certainly, the authors expressed this concern in the manuscript, but it cannot prevent readers from wondering which results are correct, the previous or the present findings. Day by day the forest areas are shrinking in Sabah, monkeys are losing their

habitats and insecticides are used abundantly to control mosquitoes. Therefore, time is a major factor in this type of results from Sabah.

Author: We thank the reviewer for their comments on clarity and have adapted the introduction and discussion sections to be more concise. We agree that it is well known that data from small scale studies cannot be extrapolated; but in the absence of more large-scale data they frequently are. Thus this work fills an important gap by providing results from a larger-scale study which will help balance/reduce overextrapolation from existing smaller studies. The data presented thus contributes to the understanding of *P. knowlesi* vector ecology outside the few sites in Kudat where studies have previously focused. We do not wish to compete with the results described in the previous studies mentioned as these were obtained through a different sampling approach (fixed sites and longitudinal) but rather compliment these with information on vector ecology beyond Kudat. We acknowledge the timing limitation of the serology component but we clearly state this in **line 598**. Although imperfect, we think the addition of serology data is useful and provides the context for transmission in this area along with vital baseline data for future studies.

Reviewer 3: Specific comments:

1. Author summary; line 57: *P. knowlesi* is not recently spilled over into human rather it is now established cause of malaria in this part of the world.

Author: 'Have altered this to state that *P. knowlesi* is a common cause of human malaria in Malaysian Borneo' (line 73).

2. The introduction and discussion sections should be shortened, clear and to the point. I think then it will be easy for international readers.

Author: Thank-you for this constructive feedback. We have now shortened the introduction by approximately 300 words.

3. There were no comments on *An. donaldi* and *An. lateus* which are vectors of *P. knowlesi* (Hanks et al. 2019; Chua et al. 2019).

Author: Thank-you for this suggestion. We do comment on *An. latens* as being a vector of *P. knowlesi* in (line 323) but too few were collected to perform a separate analysis for this species which is why we present analysis on 'An. Leucosphyrus group' mosquitoes which incorporates both *An. balabacensis* and *An. latens* individuals.

4. A yearlong study found that *An. balabacensis* is equally abundant in different habitats (Chua et al 2019). I think a comparison with this study will be helpful to understand.

Author: Thank-you for this valuable suggestion. We have now referred to Chua et al, 2019 in **line 127, 533, 534 and 562.**

5. Lines 131–138: There are several factors, such as types of agriculture, availability of mosquito breeding sites, land cover might affect the mosquito density and types. However, presence of macaques in the study area might affect the vectors specifically vectors containing *P. knowlesi.* I think data on the presence of macaques in the study areas should be added.

Author: The authors agree that this would be valuable information however is unavailable. Collecting this type of data is very labour intensive and was outwith the funding acquired for the study. We

have added a comment to the discussion on this as a point for future studies to consider (from **line 576).**

6. The results presented here is the cumulative data obtained from 4 districts, what about the data from Kudat only. Are the results only from Kudat similar as obtained in previous studies?

Author: The mosquito ecology had not been investigated before in the villages sampled in this study from Kudat district. It is not appropriate to compare vector densities with previous studies such as Wong et al, who longitudinally sampled three sites in Kudat, because these were selected based on their abundance of *An. balabacensis* to investigate seasonal changes in abundance and infection rates (we have commented on this in **line 510**). Our sampling strategy was intended to give a broad snapshot view of vectors across a wide region in Sabah. Nevertheless, if we focus in on the data generated in our study from Kudat, we can see that there is substantial heterogeneity in species and density between villages that are relatively close to one another (in one district) and of similar environmental characteristics.

7. Line 267: How Plasmodium infection was determined in mosquito by microscopy?

Author: Plasmodium infections were identified by extracting DNA from whole mosquitoes and screening using the Snounou et al (16), nested PCR method which detects DNA from all parasites belonging to the Plasmodium genus.

8. Table 1: Indicate the full name of each village.

Author: The full names of each village has now been added.

9. Were there any macaque habitats near the study sites?

Author: Macaque data was not collected as part of the study however we acknowledge the importance of this for the presence and infection rates of mosquito vectors. It is still understood that *Plasmodium knowlesi* infection occurs in vector species due to spillover from the macaque reservoir thus the presence of macaques is a significant risk factor in human infection. Collecting data on macaque presence is labour intensive and was outwith the possibility of this study due to funding constraints. We have added a comment to the discussion on this as a point for future studies to consider (from **line 576**).

10. Lines 446-447: What do you mean by this sentence?

Author: The text in lines 446-447 (now **450 – 451**) is as follows 'In these villages, the probability of trapping *An. balabacensis* per night in an HLC ranged from 0.11 – 0.42 (Fig 5A), and 0.11 – 0.50 for the Leucosphyrus group overall (Fig 5B)'. The chance of catching a *An. balabacensis* mosquito by human landing catch each night during the study period was from 0.11 – 0.42 and for all mosquitoes from the Leucosphyrus group this was 0.11 – 0.50.

11. Line 463: Why putative?

Author: We used the word putative here to describe *An. balabacensis* because it is generally considered to be the main *P. knowlesi* vector in Sabah despite studies only taking place in the Kudat district. This fact however is now out of date with a recent study published by Hawkes *et al*, (17) who found *An. donaldi* as a vector of *P. knowlesi*. We have now removed the word 'putative' from here in the manuscript.

12. The method used to determine the serology is not clear. Which antigens were used in the present study? One of my main concern in this manuscript is whether this method was validated to determine the specificity of infection at population level? Proper references are needed in this regard.

Author: The methods used to obtain measures for village level human *P. knowlesi* seropositivity are described in Herman *et al* (18) and Fornace *et al* (15). This work is referred to in lines **278-283** of the methods section.

References

1. Wong ML, Chua TH, Leong CS, Khaw LT, Fornace K. Seasonal and spatial dynamics of the primary vector of Plasmodium knowlesi within a major transmission focus in Sabah, Malaysia. PLoS Negl Trop Dis. 2015;9(10):1–15.

2. Fornace KM, Alexander N, Abidin TR, Brock PM, Chua TH, Vythilingam I, et al. Local human movement patterns and land use impact exposure to zoonotic malaria in Malaysian Borneo. Elife. 2019;8(e47602):1–17.

3. Manin BO, Ferguson HM, Vythilingam I, Fornace K, William T, Torr SJ, et al. Investigating the contribution of peri-domestic transmission to risk of zoonotic malaria infection in humans. PLoS Negl Trop Dis [Internet]. 2016;10(10):1–14. Available from: http://dx.plos.org/10.1371/journal.pntd.0005064

4. Seng CM, Matusop A, Sen FK. Differences in Anopheles composition and malaria transmission in the village settlements and cultivated farming zone in Sarawak, Malaysia. Vol. 30, Southeast Asian Journal of Tropical Medicine and Public Health. 1999. p. 454–9.

5. Brown R, Hing CT, Fornace K, Ferguson HM. Evaluation of resting traps to examine the behaviour and ecology of mosquito vectors in an area of rapidly changing land use in Sabah, Malaysian Borneo. Parasites and Vectors. 2018;11(1):1–15.

6. Hawkes F, Manin BO, Ng SH, Torr SJ, Drakeley C, Chua TH, et al. Evaluation of electric nets as means to sample mosquito vectors host-seeking on humans and primates. Parasit Vectors [Internet]. 2017;10(1):338. Available from: http://parasitesandvectors.biomedcentral.com/articles/10.1186/s13071-017-2277-3

7. Jiram AI, Vythilingam I, NoorAzian YM, Yusof YM, Azahari AH, Fong M-Y. Entomologic investigation of Plasmodium knowlesi vectors in Kuala Lipis, Pahang, Malaysia. Malar J [Internet]. 2012 Jan [cited 2014 Nov 30];11(213):1–10. Available from: http://www.pubmedcentral.nih.gov/articlerender.fcgi?artid=3476358&tool=pmcentrez&rendertype=abstract

8. Vythilingam I, Noorazian YM, Huat TC, Jiram AI, Yusri YM, Azahari AH, et al. Plasmodium knowlesi in humans, macaques and mosquitoes in peninsular Malaysia. Parasit Vectors [Internet]. 2008 Jan [cited 2014 Dec 8];1(1):1–10. Available from: http://www.pubmedcentral.nih.gov/articlerender.fcgi?artid=2531168&tool=pmcentrez&rendertype=abstract

9. Vythilingam I, Tan CH, Asmad M, Chan ST, Lee KS, Singh B. Natural transmission of Plasmodium knowlesi to humans by Anopheles latens in Sarawak, Malaysia. Trans R Soc Trop

Med Hyg [Internet]. 2006 Nov [cited 2014 Oct 14];100(11):1087–8. Available from: http://www.ncbi.nlm.nih.gov/pubmed/16725166

10. Tan CH, Vythilingam I, Matusop A, Chan ST, Singh B. Bionomics of Anopheles latens in Kapit, Sarawak, Malaysian Borneo in relation to the transmission of zoonotic simian malaria parasite Plasmodium knowlesi. Malar J. 2008;7:1–8.

11. Drakeley CJ, Corran PH, Coleman PG, Tongren JE, McDonald SLR, Carneiro I, et al. Estimating medium- and long-term trends in malaria transmission by using serological markers of malaria exposure. Proc Natl Acad Sci [Internet]. 2005;102(14):5108–13. Available from: http://www.pnas.org/cgi/doi/10.1073/pnas.0408725102

12. Hii JL, Vun YS, Chin KF, Chua R, Tambakau S, Binisol ES, et al. The influence of permethrin-impregnated bednets and mass drug administration on the incidence of Plasmodium falciparum malaria in children in Sabah, Malaysia. Med Vet Entomol [Internet]. 1987;1(4):397–407. Available from: http://www.ncbi.nlm.nih.gov/pubmed/2979556

13. Hii J, Alexander N, Chuan CK, Rahman HA, Safri A, Chan M. Lambdacyhalothrin impregnated bednets control malaria in Sabah, Malaysia. Southeast Asian J Trop Med Public Health. 1995;26(2):371–4.

14. Gregory N, Ewers RM, Chung AYC, Cator LJ. El Niño drought and tropical forest conversion synergistically determine mosquito development rate. Environmnental Res Lett. 2019;14(035003):1–8.

15. Fornace KM, Brock PM, Abidin TR, Grignard L, Herman LS, Chua TH, et al. Environmental risk factors and exposure to the zoonotic malaria parasite Plasmodium knowlesi across northern Sabah, Malaysia : a population-based cross-sectional survey. Lancet Planet Heal [Internet]. 2019;3:179–86. Available from: https://apps.who.int/iris/bitstream/handle/10665/272284/9789241565578-eng.pdf?ua=1

16. Snounou G, Singh B. Nested PCR analysis of Plasmodium parasites. Methods Mol Med [Internet]. 2002 Jan;72:189–203. Available from: http://www.ncbi.nlm.nih.gov/pubmed/12125116

17. Hawkes FM, Manin BO, Cooper A, Daim S, Homathevi R, Jelip J, et al. Vector compositions change across forested to deforested ecotones in emerging areas of zoonotic malaria transmission in Malaysia. Nat Sci Reports. 2019;9(13312):1–12.

18. Herman LS, Fornace K, Phelan J, Grigg MJ, Anstey NM, William T, et al. Identification and validation of a novel panel of Plasmodium knowlesi biomarkers of serological exposure. PLoS Negl Trop Dis. 2018;12(6):1–21.
