# Supplementary material for: Human exposure to zoonotic malaria vectors in village, farm and forest habitats in Sabah, Malaysian Borneo
Source: PLoS Negl Trop Dis. 2020 Sep 4;14(9):e0008617. doi: 10.1371/journal.pntd.0008617 (PMC7497982; doi:10.1371/journal.pntd.0008617)
Supplement: Attachment — Submitted filename: Cover letter PLOSntds.docx [file pntd.0008617.s011.docx]

| Institute of Biodiversity, Animal Health and Comparative Medicine  Graham Kerr Building  University of Glasgow  Glasgow, UK  G12 8QQ  Phone: (+44) 0141 330 4450  Fax: (+44) 0141 330 5971  Heather.Ferguson@glasgow.ac.uk  July 17th, 2020 |  |
| --- | --- |

Dear Editor,

We are very grateful to you and the reviewers for your time and consideration of our manuscript: ‘*Human exposure to zoonotic malaria vectors in village, farm and forest habitats in Sabah, Malaysian Borneo’* for publication in PLOS Neglected Tropical Diseases. In response to comments on our resubmission on 04/06/2020, we have made minor modifications to the manuscript which are detailed below:

1. L41: (2) is it a typographical error?

Early in the submission process, one of the reviewers asked for references to be added to the abstract of the manuscript. This is not common practice and there is no evidence of this being done in other published PLOSntd articles so these have been removed from this section.

2. L73: SE should be South East.

SE now changed to South East.

3. L110: Please edit km. The meaning is unclear.

This sentence on line 110 has now been edited to make the point clearer.

4. L132: What does “active” mean?

Here, the term ‘active’ infection means a current, circulating human malaria infection. We believe this is clear as it follows on from the previous sentence: ‘A particular challenge in the study of *P. knowlesi* epidemiology is that human infection rates are generally very low, thus requiring often prohibitively large sample sizes to reliably estimate prevalence.’

A new version of the manuscript (one with accepted and one with tracked changes) including these small edits has now been uploaded to the PLOS Neglected Tropical Diseases Editorial Manager.

In addition to these modifications suggested by reviewer 3, we now refer to the location of the data on line 321 which is publicly available from Harvard Dataverse. We have also made a slight change to the font size of the x and y axis text in Figure 3 and 4 and have changed the colour of the bars in Figure 3 from black to grey so that the lower end of the error bar is visible. All figures have been scanned by PACE and adjusted files have been uploaded with this submission.

We confirm that all content of this manuscript is original and has not been previously published or submitted for publication elsewhere. We declare that authors have no competing interests, and all have approved the manuscript for resubmission. Correspondence concerning this manuscript can be addressed to the lead author, Rebecca Brown (rebbrown552@gmail.com).

We look forward to your final confirmation for the publication of this article.

Kind regards,

 Rebecca Brown (on behalf of all co-authors)
